# TRAIL Induces Neutrophil Apoptosis and Dampens Sepsis-Induced Organ Injury in Murine Colon Ascendens Stent Peritonitis

**DOI:** 10.1371/journal.pone.0097451

**Published:** 2014-06-02

**Authors:** Katharina Beyer, Christian Poetschke, Lars Ivo Partecke, Wolfram von Bernstorff, Stefan Maier, Barbara M. Broeker, Claus-Dieter Heidecke

**Affiliations:** 1 Universitätsmedizin Greifswald, Klinik für Allgemein-, Viszeral-, Thorax- und Gefäβchirurgie, Greifswald, Germany; 2 Universitätsmedizin Greifswald, Abteilung für Immunologie, Greifswald, Germany; French National Centre for Scientific Research, France

## Abstract

TNF-related apoptosis inducing ligand (TRAIL) influences several inflammatory reactions by partially still unknown mechanisms. TRAIL is produced and expressed by several cells of the immune system. Murine Colon Ascendens Stent Peritonitis (CASP) represents a hyperinflammatory model of diffuse peritonitis. As we have shown previously, TRAIL strongly improves survival in murine CASP. This is accompanied by a significantly reduced infiltration of neutrophils in the associated lymphoid tissue. Additionally, it is known that TRAIL induces apoptosis in neutrophils and acceleration of neutrophil apoptosis enhances resolution of inflammatory reactions. In this study, we investigated the correlation of the protective effect of TRAIL in sepsis and its influence on neutrophils. We found that neutrophils infiltrating the lymphoid organs express the TRAIL-receptor DR5 at high density. Furthermore, we demonstrated that TRAIL-treatment enhances apoptosis of neutrophils in the spleen, lung and liver and decreases organ injury during sepsis. To further examine a role for neutrophils in TRAIL-mediated protection in CASP, we have depleted neutrophils 24 hours prior to CASP. In these depleted mice, administration of TRAIL was ineffective. We conclude that TRAIL induces apoptosis in tissue-infiltrating neutrophils thereby protecting organs from sepsis-induced injury.

## Introduction

Sepsis is a complex interplay of various cell types of the innate and the adaptive immune system. It is a dangerous consequence of the overwhelming systemic host response to extracellular bacteria and can culminate in fatal shock. Endothelial dysfunction, severe coagulopathy and hypotension with inadequate organ perfusion can result in a multi-organ dysfunction syndrom (MODS). In response to an inflammatory reaction, a compensatory anti-inflammatory response syndrome is mounted. The balance of these two forces would restore homeostasis. However, the predominance of one of those reactions is contributed to sepsis sequel [1].

Cytokines are essential mediators, which regulate inflammatory responses. During sepsis, proinflammatory cytokines are abundant in the system, especially IL6 [2] and TNF. They dominate in the acute phase of sepsis and are the main causative agents for hyperinflammation [3]. In the adjacent compensatory phase, anti-inflammatory cytokines like IL10 are secreted [3]. This anti-inflammatory reaction is further characterized by low levels of circulating lymphocytes, increased lymphocyte apoptosis and a shift from T helper 1 to T helper 2 subpopulations [4], [5].

TNF-related apoptosis inducing ligand (TRAIL) is a type II membrane protein and is expressed by several types of leukocytes during sepsis, including neutrophils, lymphoctes, macrophages and monocytes [6], [7]. This member of the TNF superfamily is known to induce apoptosis in transformed cells, but TRAIL also has an impact on inflammatory reactions. For example, TRAIL can accelerate neutrophil apoptosis [8]. Furthermore, TRAIL influences the activation of T cells [9]. Additionally, it has an impact on NK mediated killing [10], [11]. The role of TRAIL in sepsis has been insufficiently investigated. We have previously shown, that TRAIL-treatment was protective in murine Colon Ascendens Stent Peritonitis (CASP), a model of polymicrobial intraabdominal sepsis [6], as it led to significantly prolonged survival. This was accompanied by a strong decrease in neutrophil-influx in the spleen and the lymph nodes and a general prevention of apoptosis.

Neutrophils are rapidly activated in sepsis. Those cells classically provide the first line of defence against invading pathogens [12]. They are armed with reactive oxidant intermediates, proteases, antimicrobial peptides and lactoferrin to name a few. Undergoing a special form of cell death, they generate neutrophil extracellular traps (NETs) [13]. Hence, neutrophils are essential for the initial control of microbial invasion. However, their systemic activation in sepsis leads to massive tissue damage. Although controlled termination of this acute inflammatory response is critical to restrict tissue damage and provide tissue repair [13], during infection or other major stressors granulopoesis, neutrophil survival and circulating neutrophil counts can increase dramatically [13]. Additionally, such conditions modulate the way of neutrophil death [13]. At sites of inflammation neutrophils undergo apoptosis and this can be induced by TNF, FasL or TRAIL [8]. Apoptotic neutrophils are phagocyted by macrophages and this leads to resolution of inflammation [14]. Furthermore, phagocytosis of neutrophils by macrophages decreases IL-23 production and downstream IL-17a secretion by T cells resulting in a limitation of granulopoesis [13]. However, bacterial products like LPS and pro-inflammatory cytokines delay neutrophil apoptosis [13] [15], which further contributes to uncontrolled tissue damage [16]. Hence, whereas in general prevention of apoptosis has been shown to be protective in murine sepsis models [17], the specific delay of apoptosis of neutrophils can be harmful in sepsis [18], [19].

It is known that TRAIL promotes apoptosis of senescent neutrophils [20]. Over the last years, evidence has been accumulated that TRAIL can interact with neutrophils in models of hyperinflammation thereby promoting the resolution of inflammation [8], [15], [21]. In this study we have investigated the role of neutrophils in the context of TRAIL-mediated protection in a murine sepsis-model.

## Materials and Methods

### Mice

Female 8- to 12-weeks old C57BL/6 mice (weight 20–25 g) were purchased from Charles River Laboratories (Sulzfeld, Germany). Mice were housed in a conventional animal facility and were allowed to adapt for at least 2 weeks. During all experiments, mice were provided with food and water ad libitum. A 12-hour light/12-hour dark cycle was used. Mice were housed in groups of five animals per cage. All experiments were performed according to German animal safety regulations and were approved by the “Landesamt für Landwirtschaft, Lebensmittelsicherheit und Fischerei Mecklenburg-Vorpommern”, Germany (AZ 7221.3-1.1-017/12).

### Cell Culture Experiments

Mice were sacrificed by cervival dislocation under deep anaesthesia. Spleens were taken and splenocytes were isolated by passing the organs through a 100 µm nylon mesh (BD falcon cell stainer; BD bioscience). Cells were stored on ice and washed three times with 2% fetal calf serum in PBS. Cells were maintained in RPMI-1640 medium supplemented with 10% fetal calf serum, 100 U/ml of penicillin and 100 µg/ml of streptomycin. Tissue culture reagents were obtained from Gibco (Invitrogen, Carlsbad, California, USA). Cell cultures were kept in a humidified incubator at 37°C with 5% CO_2_.

#### *In vitro* TRAIL-treatment

Recombinant soluble mouse TRAIL (Biomol GmbH, Hamburg, Germany) (purity 95%, endotoxin level <1.0 EU per 1 g protein) was dissolved in sterile RPMI-1640 medium. Cells were incubated with respective stimulants for 48 hours. Recombinant TRAIL was used at a final concentration of 100 ng/ml and E. coli-LPS (Sigma Aldrich, Taufkirchen, Germany) at a final concentration of 1 µg/ml.

Cells were harvested at the respective time points, washed three times and were finally resuspended in PBS. The cell count was determined using a CASY cell counter (Roche, Mannheim, Germany). After that, cell suspensions were analyzed by appropriate FACS analyses or a cell viability assay as described below.

#### Cell viability assay

A Cell Titer Blue Assay (Promega, Mannheim, Germany) was performed according to manufacturer’s instructions. In brief, Cell Titer Blue substrates were added and plates were incubated for 4 h at 37°C. Subsequently, fluorescence (excitation at 544 nm and emission at 590 nm) was measured on a plate reader. Triplicates were run for each measurement and means were calculated. Controls without cells were run in parallel.

### CASP Surgery

CASP surgery was performed as previously described [22]. In brief, mice were intraperitoneally anesthetized with Ketamin (100 µg/g (wt/wt)) and Xylazin (8 µg/g (wt/wt)). The ascending colon was exposed and a catheter (16- gauge; Venflon; BOC, Ohmeda AB, Sweden) was inserted through the antimesenteric wall into the lumen of the ascending colon and was fixed with two sutures (7/0 Ethilon thread; Ethicon, Norderstedt, Germany). The inner needle of the stent was removed and the stent was cut 2 mm above the puncture site. To ensure proper intraluminal positioning of the stent, stool was milked from the cecum into the stent until a small amount appeared. Fluid replacement was performed by injecting 0.5 ml of sterile saline solution into the peritoneal cavity before closing the abdominal wall (4/0 Polyester). Postoperatively, mice received buprenorphine (0.1 mg/kg (wt/wt) i.m.) in order to reduce suffering. All CASP surgery was performed by the same surgeon (KB) to minimize variance.

### *In vivo* Recombinant TRAIL Treatment and Survival Analyses

Recombinant soluble mouse TRAIL (Biomol GmbH, Hamburg, Germany) (purity 95%, endotoxin level <1.0 EU per 1 g protein) was dissolved in sterile saline at a final concentration of 0.1 g/l and given intravenously at a dose of 1 µg/g (wt/wt) 1 hour, 24 hours, and 48 hours after induction of CASP. Control animals received equal volumes of sterile saline. Survival and disease severity were monitored every three hours for 10 days. According to the sepsis severity score reported by Zantl et al. [23], disease severity was scored on the basis of general appearance, breathing frequency and spontaneous as well as provoked behaviour. Scoring points reaching from 0 (healthy) to 3 (severe alterations) were assigned to every mouse for each issue. Mice reaching a severity score that indicated a disease point of no return were euthanized by cervical dislocation under deep anaesthesia.

### Depletion of Neutrophils

Neutrophils were depleted as described before [18], [24]. In brief, 25 µg/g (wt/wt) of a functional grade purified monoclonal anti-mouse Ly-6G antibody was given intravenously 24 h before induction of CASP. Control animals received 25 µg/g (wt/wt) of an isotype control (rat IgG2bk, eBioscience). Depletion of neutrophils was confirmed within the blod by FACS analyses every 3 hours until 48 hours after induction of CASP. Neutrophils were detected via expression of CD11b+Ly6Cmid.

### FACS Analyses

Mice were sacrificed 20 hours after induction of CASP by cervival dislocation under deep anaesthesia. Spleens were taken and splenocytes were isolated. After blocking with FcBlock (BD Biosciences, San Jose, California, USA) cells were stained with appropriate antibodies according to the manufacturer’s instructions. PE-conjugated anti-mouse DR5 and PE-conjugated anti-mouse TRAIL were purchased from eBioscience. Isotype controls (PE-conjugated Armenian Hamster IgG and Rat IgG2ak) were also purchased from eBioscience. APC- and FITC-conjugated anti-Ly6G (clone 1A8) was purchased from Miltenyi (Miltenyi Biotec GmbH, Bergisch Gladbach, Germany). Rat IgG2a (Miltenyi) was used for isotype control. FITC-conjugated anti-B220, FITC-conjugated anti-CD3, PE-conjugated anti-CD11b and FITC-conjugated anti-Ly6C was purchased from Pharmingen (BD Biosciences, San Jose, California, USA). Appropriate isotype controls were also purchased from Pharmingen.

FACS analysis was done using a BD FACS Calibur system. For cell analysis FlowJo (Treestar Inc., Ashland, USA) and WinMDI software were used.

### Immunohistochemistry

#### Staining of TRAIL

Organs were harvested 20 h after induction of CASP followed by overnight fixation in 4% paraformaldehyde at 4°C. Fixed organs were paraffin-embedded and cut into 4-µm slices. Slides were rehydrated followed by antigen retrieval through boiling in 1 mM citric acid buffer (pH 6.0). Endogenous peroxidases were inactivated by adding 1,5% hydrogen peroxide. Nonspecific binding sites were blocked using appropriate blocking reagents. Subsequently, slides were incubated with primary antibodies overnight at 4°C. Polyclonal goat anti-TRAIL (Santa Cruz Biotechnologies, Heidelberg, Germany) was used as the primary antibody. Primary antibodies were detected using a peroxidase-conjugated secondary antibody (GE Healthcare, California, USA) in combination with a DAB detection kit (Pierce, Rockford, USA). The number of TRAIL-positive cells in four high-power fields (HPFs, 200x magnification) was counted using an Olympus IX70 Microscope (Olympus America Inc., Center Valley, PA). The mean was calculated.

#### Staining of apoptotic cells and neutrophils

For detection of apoptotic neutrophils, spleens, lungs and livers of TRAIL-treated and saline-treated septic control mice were taken 20 h after induction of CASP. Organs were snap frozen in liquid nitrogen and fresh frozen sections were cut at 6 µm and fixated in ice-cold acetone for 10 minutes. Apoptosis was detected by terminal deoxynucleotidyl transferase dUTP nick end labeling (TUNEL) staining using an ApopTag Fluorescein apoptosis detection kit (Chemicon International, Temecula, CA) according to manufacturer’s instructions. To visualize neutrophils, counterstaining was performed by incubating sections with rat anti-Ly6G (clone RB6-8C5, rat IgG2a kappa, BD Pharmingen) and an appropriate FITC-labelled secondary antibody (FITC mouse anti-rat IgG2b, BD Pharmingen). Slides were analysed by confocal laser scanning microscopy using an Olympus IX70 microscope (Olympus America Inc., Center Valley, PA). Overlay pictures of the green and the red channels were generated. For each slide the number of neutrophils (green), apoptotic cells (red) and apoptotic neutrophils (yellow) was counted in three high-powerfields (HPFs) and the mean was calculated. The ratio of apoptotic neutrophils over all neutrophils and the fraction of apoptotic neutrophils to all apoptotic cells were calculated.

### Serum Analysis

CASP was induced and TRAIL-treatment was performed as described above. 20 h after induction of CASP mice were sacrificed by cervical dislocation. Blood samples were collected via puncture of the orbital plexus. The blood was allowed to clot. After centrifugation at 300 g the serum was collected. Levels of alanine aminotransferase (ALAT), aspartate aminotransferase (ASAT) and creatinine were quantified using a standard clinical automatic analyzer.

### Statistical Methods

Survival analyses were performed using the Kaplan-Meier method. Differences in survival were analyzed by the logrank test. Continuous variables were compared by the Kolmogorov Smirnov Test for two independent groups and by the Kruskal Wallis test for more than two independent groups. For all calculations SPSS software (SPSS, edition 21.0, Chicago, USA) was used. A p-value of less than 0.05 was considered statistically significant. Data are expressed as mean ± standard deviation.

## Results

### TRAIL-treatment Did Not Alter the Viability of Splenocytes *In vitro*

**Figure 1:**
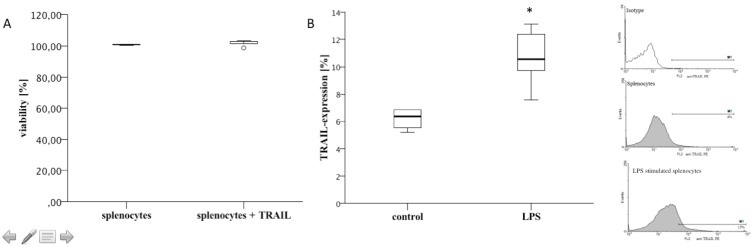
TRAIL-treatment did not influence cell viability *in vitro*. LPS-stimulation increased TRAIL-expression by splenocytes. A: Cultures of splenocytes were stimulated with TRAIL (100 ng/ml) for 48 hours. Cell viability was determined using a CellTiter Blue Assay. Box plots and outliers are depicted. TRAIL-stimulation did not alter the viability of splenocytes. n = 5/group; results are representative of two independently performed experiments. B: Cultures of splenocytes were stimulated with LPS (1 µg/ml) for 24 hours. TRAIL-expression was determined by FACS analyses. Isotype controls were used for background staining. Box plots and representative histograms of FACS analyses are shown. LPS stimulation significantly increased the expression of TRAIL on the cell surface of splenocytes. One of two experiments in which similar results were obtained is shown. *: p<0.05.

To exclude an induction of any type of cell death through a direct interaction of TRAIL with non-septic immune cells, splenocytes were stimulated with recombinant TRAIL for 48 hours. Using a cell viability assay, we could show that TRAIL treatment did not alter the viability of splenocytes (100+−2% viability in non-stimulated splenocytes versus 101+−6% in TRAIL-stimulated cultures, n = 5/group, figure 1A).

### LPS-simultation Increased the Expression of TRAIL on the Cell Surface of Splenocytes *In vitro*

Stimulation of splenocytes with LPS for 24 hours led to an increased TRAIL-expression on the cell surface: As determined by FACS analyses, TRAIL-expression was 6.4+−0.4% in non-stimulated splenocytes. In marked contrast, TRAIL-expression increased to 11+−0.8% in LPS-stimulated cells (n = >4, p = 0.08, figure 1B).

### TRAIL was Predominantly Expressed within the Red Pulp of Septic Mice

**Figure 2:**
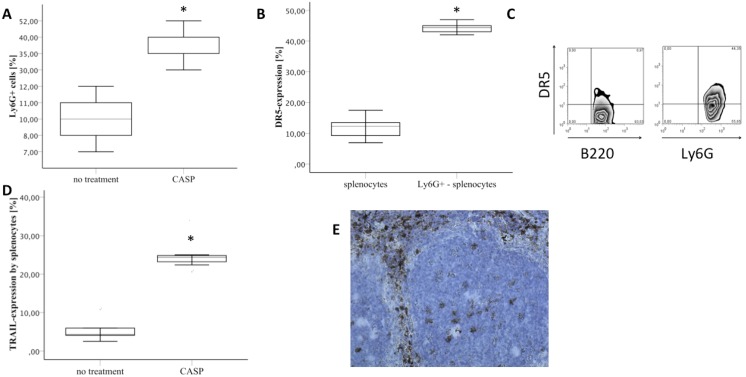
CASP led to increased expression of DR5 and TRAIL. DR5 was predominantly expressed on neutrophil cell surface. A: The fraction of neutrophils within the spleen was determined by FACS analyses in non-treated mice (no treatment) versus septic mice 20 hours after induction of CASP (CASP). Box plots are shown. n = 5/group. Results are representative of three independent experiments. B: DR5-expression as well as Ly6G-expression by murine splenocytes was determined by FACS analysis (n = 5). The expression of DR5 on the cell surface of Ly6G+ -splenocytes was compared to the expression of DR5 on all splenocytes. Boxplots are shown. Isotype controls were used for background staining. One of two experiments in which similar results were obtained is shown. C: Representative FACS density plots of the expression of DR5 by splenocytes are shown. Plots were gated on B220 and Ly6G respectively. D: TRAIL-binding on the cell surface of splenocytes is shown as determined by FACS analysis of spleens of untreated and septic mice (CASP; 20 h after CASP) (n = 5/group). Isotype controls were used for background staining. Box plots and outliers are depicted. TRAIL-expression was significantly increased during CASP. Results are representative of three independent experiments. E: TRAIL was stained in spleens of septic TRAIL-treated mice via immunohistochemistry. TRAIL was mainly detected in cells of the splenic red pulp (brown coloured cells, n = 5). One representative picture of five is depicted. A 200x magnification is shown. *p<0.05.

As determined by FACS analyses, TRAIL-binding of splenocytes was negligible in non-septic mice. However, 20 hours after CASP, TRAIL was significantly upregulated (5.5%±3.2% versus 24.3%±2.5%, p = 0.001) (figure 2D). As shown by immunhistochemistry, TRAIL+ cells were mainly located within the red pulp nearly sparing the white pulp nearly completely (figure 2E).

### DR5 (Death Receptor 5, TRAIL Receptor 2) was Strongly Expressed by Neutrophils

The expression of DR5 – the only TRAIL-receptor with a functional death domain in mice - was determined in spleens of untreated mice. As shown by FACS analysis the overall expression of DR5 within the spleen was 11.8%±4.0% (n = 5). The expression of DR5 by B-cells (B220 positive) and T-cells (CD3 positive) of the spleen was about 10% each. In contrast, neutrophil expression of DR5 was 44.3%±1.9% (n = 5) (figure 2B+C). Therefore, the expression of DR5 within the spleen is highest in splenic neutrophils.

### Exogeneous TRAIL Led to a Decrease of Overall Apoptosis but Increased Neutrophil Apoptosis in Septic Organs

**Figure 3:**
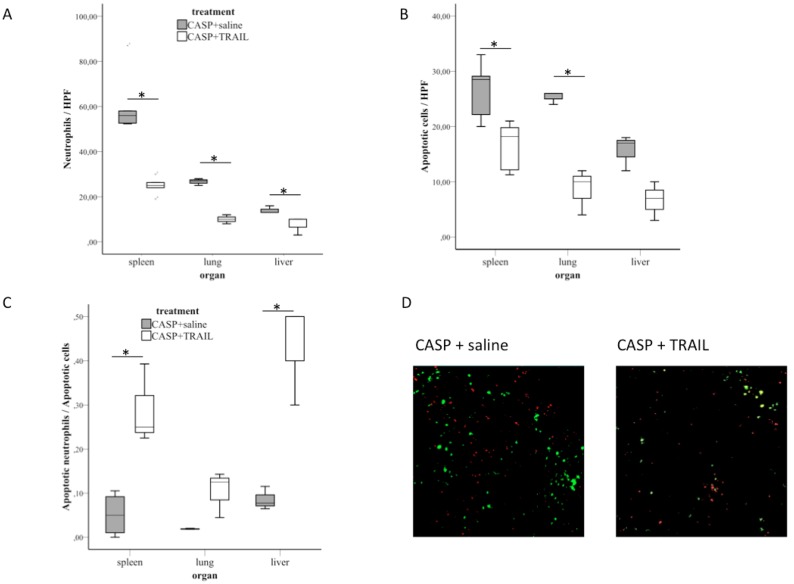
TRAIL-treatment led to induction of apoptosis in neutrophils in sepsis. A) Spleens, livers and lungs of septic saline-treated (CASP+saline) and septic TRAIL-treated (CASP+TRAIL) were analyzed 20 h after induction of CASP. Sections were stained for Ly6G. Ly6G-positive cells of respective organs (n = 5/group for each organ) were counted in three HPFs and the mean was calculated. The number of neutrophils per HPF is depicted. Box plots and outliers are shown. The infiltration of neutrophils within the septic organs is significantly decreased by TRAIL-treatment in sepsis. Results are representative of two independent experiments. B) The number of apoptotic cells within the spleen, liver and lungs was determined by immunohistochemistry (n = 5/group for each organ, mean of 3 HPFs). TUNEL-straining was performed 20 hours after CASP. Box plots and outliers are shown. TRAIL-treatment decreased the number of apoptotic cells. Results are representative of two experiments performed independently. C) Apoptotic neutrophils were detected by staining Ly6G and TUNEL. The number of apoptotic neutrophils within the respective septic organs 20 hrs after induction of CASP was counted in three HPFs and the mean was calculated (n = 5/group for each organ). Additionally, the number of total apoptotic cells per HPF was counted. The ratio of apoptotic neutrophils over all apoptotic cells was calculated for each HPF. Box plots and outliers are depicted. TRAIL-treatment increased the fraction of apoptotic neutrophils 20 hrs after induction of CASP within the septic organs. D) Representative immunohistochemical analysis of Ly6G (green) and TUNEL (red) in spleens of septic mice 20 hrs after induction of CASP with (right) and without (left) TRAIL-treatment. Apoptotic neutrophils appear yellow. *p<0.05.

CASP leads to an influx of neutrophils into the spleen: As shown by FACS analyses, the number of neutrophils within the spleen was 9.6+−0.9% in untreated mice. In marked contrast, CASP led to a significant increase of this number (38.4+−3%, p = 0.01, n = 5/group, figure 2A). As shown by immunohistochemistry there were 61.2±14.6 neutrophils per HPF within the spleen 20 h after induction of CASP. TRAIL-treatment reduced the number to 24.9±3.0 neutrophils per HPF (n = 5, p = 0.04) (figure 3A).

This effect was also evident in septic livers and lungs of TRAIL-treated animals: Whereas there were 26.6+−0.8 neutrophils per HPF in lungs of saline-treated mice, TRAIL-treatment reduced the number to 10.0+−1.0 (p = 0.01). This was also true for livers of saline-treated and TRAIL-treated animals (14.0+−1.0 in saline-treated septic animals versus 7.6+−2.3 in TRAIL-treated septic animals, p = 0.06).

Therefore, TRAIL-treatment decreased the number of neutrophils in the septic organs.

Additionally, TRAIL-treatment reduced apoptosis of splenic cells to near one half (from 26.6+−5.3 to 16.5+−4.4/HPF, p = 0.01) in septic mice (figure 3A). The same effect was observed in lungs (25.3+−0.6/HPF in saline-treated animals versus 8.6+−1.4/HPF in TRAIL-treated animals, p = 0.015) and within the liver (15.6+−1.9/HPF in saline-treated animals versus 6.6+−2.0/HPF in TRAIL-treated animals) of saline-treated and TRAIL-treated septic mice (figure 3B).

To assess the number of apoptotic neutrophils within the septic organs, TUNEL stained sections were counterstained with anti-Ly6G. TRAIL-treatment led to induction of apoptosis of neutrophils in septic spleens causing an overall decrease in the number of neutrophils. In saline-treated animals 5.1%±2.1% of all apoptotic splenic cells were neutrophils. This fraction increased to 28.9%+−9.0% in TRAIL-treated septic mice (p = 0.03, n = 5/group, figure 3C, D). This effect was also observed in livers and lungs of saline- and TRAIL-treated septic animals: Whereas 1.8+−0.0% of all apoptotic cells were neutrophils in saline-treated septic lungs, this number increased to 10.4+−3.0% in TRAIL-treated septic lungs (p = 0.1, figure 3D). Additionally, TRAIL-treatment led to an increase in the fraction of apoptotic neutrophils within the liver (8.5+−1.5% versus 43.4+−6.6%, p = 0.03, figure 3C).

### TRAIL Treatment Decreased Tissue Injury in Sepsis

**Figure 4:**
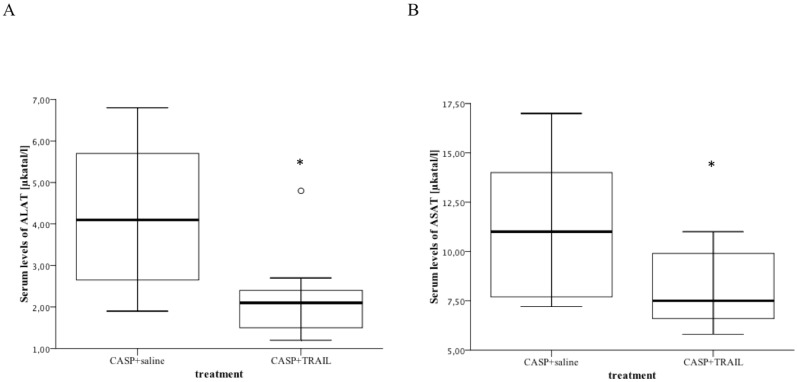
The levels of ALAT and ASAT were significantly decreased in TRAIL-treated septic mice. CASP with and without TRAIL-treatment was performed as described above. Mice (n = 5/group) were sacrificed 20 hours after induction of CASP. Serum samples were taken and serum levels of ALAT and ASAT were determined. Results are depicted as box plots of serum levels of ALAT (A) and ASAT (B). TRAIL treatment significantly decreased the serum levels of ALAT and ASAT in sepsis. *p<0.05.

Liver and kidney failure crucially contribute to septic multi organ failure. Serum levels of ALAT and ASAT as well as creatinine levels were used as organ function markers. TRAIL-therapy significantly decreased the levels of ALAT (2.2±0.4 versus 4.2±0.7, p = 0.04, n = 5) and ASAT (8.1±0. versus 11.3±1.5, p = 0.049) in sepsis (figure 4). Similarly, serum creatinine levels were reduced in TRAIL-treated septic mice when compared to saline-treated septic mice.

### Depletion of Neutrophils 24 Hours before CASP Induction Decreased Sepsis Survival and Abolished the Therapeutic TRAIL Effects

**Figure 5:**
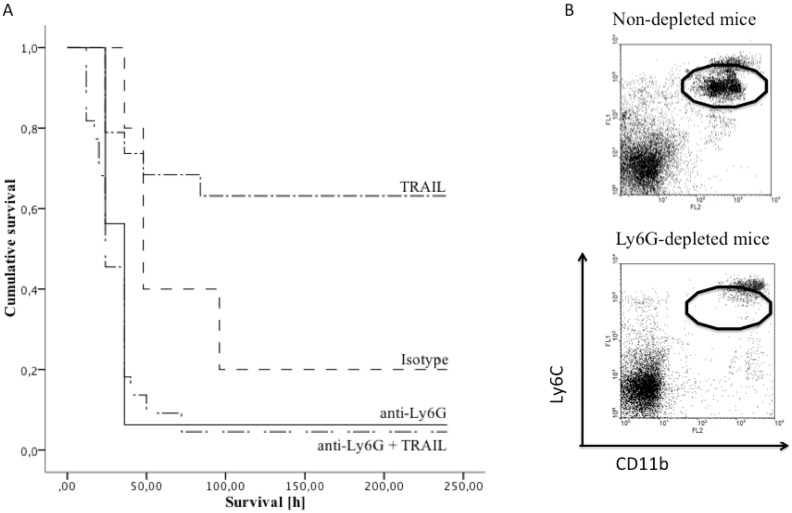
TRAIL-treatment improved survival of CASP. This effect was abrogated by depleting neutrophils. A: Survival of CASP is depicted as Kaplan Meier curves. Mice were treated with anti-Ly6G 24 hrs before CASP induction (anti-Ly6G) to deplete neutrophils. Controls received appropriate isotype controls (isotype). Neutrophil-depleted (anti-Ly6G, TRAIL) and untreated mice (TRAIL) received TRAIL (1 µg/g (wt/wt)) 1 h, 24 h and 48 h after CASP intravenously. TRAIL treatment significantly improved survival of sepsis in previously untreated mice (p<0.001). However, TRAIL-treatment was ineffective in Ly6G-depleted mice. B: Depletion of neutrophils was confirmed via FACS analyses. Representative data 48 hrs after neutrophil depletion are shown. The oval indicates neutrophils detected via CD11b+Ly6Cmed expression.

Depletion of neutrophils 24 hours before CASP led to significantly decreased survival of sepsis, which was not restored by TRAIL-treatment. Mice were treated with anti-Ly6G 24 h before CASP, which led to successful induction of neutropenia up to 48 h after CASP (flow cytometry, figure 5B). After induction of CASP, isotype control treated mice showed a mean survival of 93.6 h (survival rate 20%, n = 10) whereas the neutrophil depleted group displayed a mean survival of only 43.5 h (survival rate 6.2%,) (p = 0.03, n = 13/group) (figure 5A). While TRAIL-treatment improved survival in isotype-treated controls, the effect was completely abrogated in Ly6G-depleted mice (p = 0.6, n = 10) (figure 5A).

## Discussion

Despite recent advances in the understanding of TRAIL’s functions, the discussion about the therapeutic benefits of TRAIL in sepsis is still ongoing [6], [15], [25]. As we have shown earlier, TRAIL improves survival of sepsis in Colon Ascendens Stent Peritonitis (CASP) [6]. In contrast, other groups have shown detrimental effects of TRAIL in other inflammatory settings [25], [26]. The group of Gurung has demonstrated TRAIL-dependent immune suppression following sepsis. In their hands TRAIL impaired the outcome of secondary bacterial infection [25]. The authors reported that this effect depended on CD8+ cells and they conclude that TRAIL-expressing regulatory CD8+ cells are causally related to immune unresponsiveness after sepsis [25].

In our study of Colon Ascendens Stent Peritonitis, TRAIL was strongly protective. Following TRAIL-treatment in septic mice, TRAIL was presumably bound by the numerous DR5-receptor-bearing cells located in the splenic red pulp. On the other hand, the white pulp was nearly free from TRAIL-binding cells. This suggests that lymphocytes may not be the main players in the TRAIL-mediated protection during CASP.

As we have shown before, CASP represents a model of diffuse peritonitis, which is accompanied by a strong and continuously increasing hyperinflammatory reaction [22]. In marked contrast, cecal ligation and puncture (CLP) mimics an intraabdominal abscess leading to the continuous release of much lower amounts of bacteria and cytokines [22]. It is thus conceivable that mechanisms of protection differ between CASP and CLP. In CASP it is crucial to regulate the overwhelming hyper-inflammation, whereas in CLP limiting the immune suppression may be more decisive [27]. Such a scenario could explain why the anti-inflammatory TRAIL effects turned out to be beneficial in CASP but detrimental in CLP.

Neutrophils are powerful immune effector cells, whose aggressive potential must be tightly regulated to minimize tissue damage. Hence, they are sensitive to numerous proapoptotic stimuli like TNF, FasL and TRAIL in different contexts: Neutrophils migrating to sites of inflammation appear to become resistant to Fas- and TNF-induced apoptosis [28]. This anti-apoptotic effects are triggered by bacterial products like LPS and proinflammatory cytokines [13] and are essential for the increased survival of neutrophils during sepsis. Young freshly isolated neutrophils are TRAIL-resistant, whereas senescent neutrophils can become sensitive to TRAIL-mediated apoptosis [20]. However, there is ongoing discussion that TRAIL might have an important role for apoptosis of tissue neutrophils in inflammation [20]. In this study, we could show that the fraction of apoptotic neutrophils in septic organs is significantly increased following TRAIL-treatment. These results suggest that TRAIL induces apoptosis of tissue neutrophils in CASP and thus dampens hyperinflammation. This limits undirected neutrophil activity and tissue damage.

As we have shown before [6], TRAIL-treatment decreased the number of infiltrating neutrophils in the lymphatic organs during CASP. Furthermore, TRAIL-treatment decreased the number of apoptotic cells within the lymphatic organs during CASP [6]. The present study confirms and extends this observation. TRAIL-treatment significantly increased the number and proportion of apoptotic neutrophils in the spleen, while abrogating apoptosis in other immune cells [6]. In the present study, we detected apoptotic cells using a TUNEL assay. Bearing in mind that there are concerns regarding the specificity of TUNEL assays in distinguishing between apoptotic and necrotic cells especially in cases of predominantly necrotic injury [29], we have previously confirmed the decreased rates of apoptotic cells following TRAIL-treatment through propidium iodide cell cyclus analyses [6].

Our observations are well compatible with the notion that in CASP TRAIL promotes apoptosis in tissue neutrophils thereby limiting the overshooting activity of these cells, dampening inflammation and reducing tissue damage. In support of this idea TRAIL-treatment of septic mice reduced the CASP-related increases of serum levels of ASAT, ALAT and creatinine reflecting less tissue damage in these essential organs.

Mc Grath et al. have described that TRAIL-deficiency enhances the inflammatory response in LPS-induced lung injury and in zymosan-induced peritonitis [15]. Without TRAIL, neutrophil numbers were increased and neutrophil apoptosis reduced. These effects could be reversed by administration of recombinant TRAIL [15]. This effect was dose-dependent, since administration of a supra-physiologic dose of TRAIL further decreased inflammation and promoted neutrophil apoptosis. These data are fully in line with our results.

Lum et al. have shown that TRAIL induced apoptosis in senescent neutrophils whereas young mature neutrophils were TRAIL-resistant. Furthermore, only senescent neutrophils became TRAIL-positive [20]. These results are supported by our findings that the viability of splenocytes has not been affected by TRAIL-treatment *in vitro*. In the first phase of sepsis neutrophils are the first-line defence against bacteria. The data of Lum et al. suggest that these young mature neutrophils are TRAIL-resistant and do thus not undergo apoptosis following TRAIL-treatment. As we have reported earlier [6], we have found increased numbers of neutrophils within the peritoneal lavage of TRAIL-treated mice 12 hours after induction of CASP. Obviously, TRAIL enhances infection control in this phase of sepsis through yet unknown mechanisms. As hyperinflammation increases, the termination of inflammation becomes more and more important [19]. In this stage of sepsis, neutrophils presumably become TRAIL-sensitive. Therefore, TRAIL-treatment limits hyperinflammation and thereby increases survival of CASP.

In summary we propose the following model: TRAIL is protective during distinct early stages of peritonitis: In the very early phase, TRAIL improves infection control by enhancing the accumulation of effector cells within the peritoneum. As hyperinflammation increases, neutrophils undergo apoptosis upon TRAIL-stimulation. In this phase of peritonitis, the protective effect is due to the regulation of overshooting neutrophil effector mechanisms. The later phase of sepsis is characterized by immune suppression. In this context TRAIL may be detrimental by deepening the T cell anergy. Considering the dual role of TRAIL in sepsis, close monitoring of the sepsis stage would be a prerequisite for its potential therapeutic application in the clinics.
